# Clinical Pathological Features and Carcinogenic Risk Factors of Colorectal Lateral Spreading Tumors With Skirt Features

**DOI:** 10.1155/cjgh/9920606

**Published:** 2025-06-11

**Authors:** Longping Chen, Junguo Chen, Linfu Zheng, Jin Zheng, Binbin Xu, Dazhou Li, Wen Wang

**Affiliations:** ^1^Department of Gastroenterology, Fuzong Clinical Medical College of Fujian Medical University, Fuzhou 350025, China; ^2^Department of Gastroenterology, 900th Hospital of PLA Joint Logistic Support Force, Fuzhou 350025, China; ^3^Department of Gastroenterology, Dongfang Hospital of Xiamen University, School of Medicine, Xiamen University, Fuzhou 350025, China; ^4^West China Xiamen Hospital of Sichuan University, Xiamen 361000, China

**Keywords:** carcinogenesis, lateral spreading tumors, skirt

## Abstract

**Objective:** This study aims to investigate the clinical pathological features of colorectal lateral spreading tumors (LSTs) with skirt features and the associated carcinogenic risk factors.

**Methods:** A total of 390 cases of colorectal LSTs, treated via endoscopy at the Digestive Endoscopy Center of the 900th Hospital of the People's Liberation Army Joint Logistics Support Force between January 2021 and August 2023, were included. The cases were categorized into a skirt group (30 cases) and a group without a skirt (360 cases) based on the presence of skirt features. The clinical pathological characteristics, including age, gender, endoscopic features (lesion diameter, location, morphology), and histological types, were compared between the two groups. Additionally, the risk factors for carcinogenesis in LSTs with a skirt were analyzed.

**Results:** Among the 390 LSTs cases, 30 (7.69%) exhibited skirt features, with 23 lesions located in the rectum (76.67%) and 26 lesions having a diameter of ≥ 20 mm (86.67%). Histological classification revealed 10 cases (33.33%) of adenomas with low-grade intraepithelial neoplasia (LGIN), 9 cases (30.00%) of adenomas with high-GIN (HGIN), and 11 cases (36.67%) of carcinoma. The proportions of female patients, rectal lesions, lesions with a diameter of ≥ 20 mm, mixed nodular lesions, and those classified as carcinoma were significantly higher in LSTs with a skirt group compared to LSTs without a skirt group. Rectal lesions (*p*=0.001, OR = 8.588, 95% CI: 2.428–30.379) and lesion diameters ≥ 20 mm (*p*=0.008, OR = 4.538, 95% CI: 1.477–13.940) were identified as independent predictors of skirt presence in colorectal LSTs. Age ≥ 60 years (*p*=0.002, OR = 22.667, 95% CI: 3.140–163.629) was found to be an independent risk factor for carcinogenesis in LSTs with a skirt.

**Conclusion:** Compared with LSTs without a skirt, the results indicated that LSTs with a skirt are more commonly found in female patients, predominantly has a diameter of ≥ 20 mm, typically presents as a mixed nodular type, is frequently located in the rectum, and is often classified as carcinoma. The presence of rectal lesions and lesion diameter ≥ 20 mm increases the likelihood of skirt features in LSTs. Furthermore, advanced age (≥ 60 years) may elevate the risk of carcinogenesis in LSTs with a skirt, necessitating thorough preoperative assessments and complete resection during endoscopic removal of such lesions.

## 1. Introduction

In 2021, there were 5.26 million new cases of gastrointestinal cancer worldwide, with approximately 3.7 million deaths, among which colorectal cancer posed the most significant global burden. The incidence of colorectal cancer has been steadily increasing in recent years, but most patients are already in the middle or advanced stages at the time of diagnosis [1]. Colorectal cancer typically follows a normal epithelium-adenoma-carcinoma sequence, with the progression from ordinary raised-type adenomatous polyps to tumors generally taking 10 years. However, lateral spreading tumors (LSTs) can progress to colorectal cancer in just 3 years, with an extraordinarily high malignant transformation rate ranging from 8.4% to 52.5% [2].

LSTs are superficial lesions greater than 10 mm in diameter that spread laterally rather than vertically. These tumors have unique morphological characteristics, special growth patterns, and a relatively high malignant potential, making them a precancerous lesion that is easily overlooked in colorectal cancer [3]. “Skirt” lesions refer to a specific type of micro-elevated lesion at the edges of LSTs, characterized by a flat shape and wide glandular openings (pits), which are prone to being missed in ordinary white light endoscopy. Skirt lesions have the potential to transform into LSTs from normal mucosal tissue and may play a special role in the early stages of LSTs carcinogenesis [4]. According to an international study, LSTs with skirt lesions account for about 3.42% of all LSTs. Although there are many reports on LSTs, studies specifically focused on LSTs with skirt lesions are relatively scarce. Given the high rate of missed diagnosis and their potential for malignancy, this study aims to retrospectively analyze the clinical and pathological features of skirt lesions versus without skirt lesions, explore the factors influencing the occurrence of skirt lesions, and assess their impact on carcinogenesis, with the goal of enhancing clinical awareness and improving early diagnosis and treatment of LSTs with a skirt.

## 2. Materials and Methods

### 2.1. Patients

A total of 390 patients diagnosed with colorectal LSTs between January 2021 and August 2023 at the Digestive Endoscopy Center of the 900th Hospital of the Joint Logistics Support Force of the Chinese People's Liberation Army were included.

Inclusion criteria: (1) Patients diagnosed with LSTs via colonoscopy at our hospital; (2) Patients who underwent endoscopic or surgical treatment. All endoscopic procedures were performed by senior endoscopists, they had more than 5 years of experience in pigment endoscopy and had performed over 300 magnification endoscopy procedures. Additionally, all patients with LSTs underwent indigo carmine staining or magnification endoscopy prior to surgery to evaluate the lesions and their surrounding margins. Endoscopic treatments included EMR (122 cases) and ESD (251 cases), while 17 cases underwent radical surgery for colorectal cancer. Of the 251 ESD cases, 229 (91.24%) had negative horizontal and vertical resection margins, achieving curative resection; 11 had positive horizontal margins, 1 required additional surgical intervention; 10 had positive vertical margins, including 4 cases of SM1 and 6 of SM2, 5 of which required further surgical treatment.

**Figure 1:**
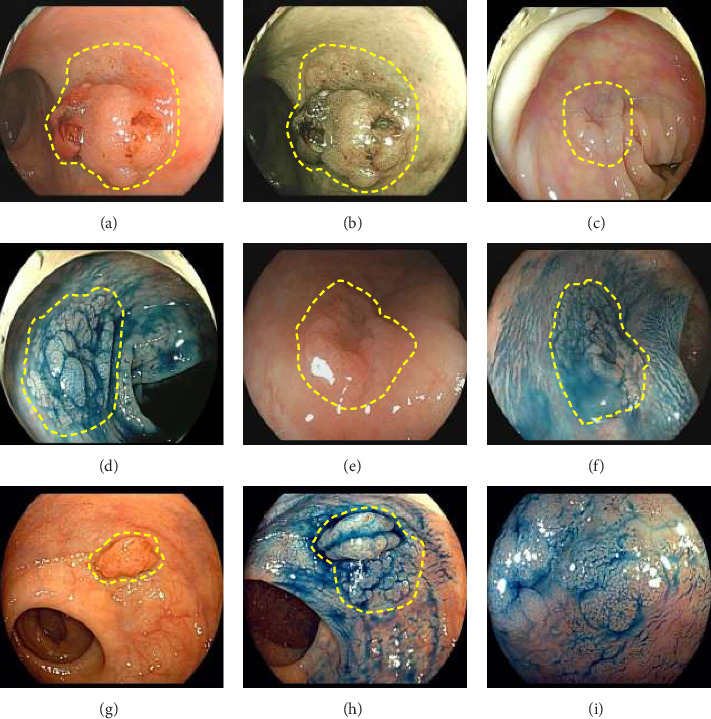
LSTs with skirt lesions. (a) Skirt lesions under white light endoscopy. (b) Skirt lesions under NBI mode. (c, e, g) Unstained skirt lesions. (d, f, h) Indigo carmine-stained skirt lesions. (i) Magnified endoscopy of indigo carmine-stained skirt lesions.

Exclusion criteria: (1) patients with inflammatory bowel disease or familial adenomatous polyposis; (2) patients with incomplete clinical data. The subjects were divided into two groups: the LSTs with a skirt group (30 cases) and the LSTs without a skirt group (360 cases). The skirt structure is shown in Figure 1, defined as a type of micro-elevated lesion around the LSTs presenting a flat shape under magnified endoscopy or indigo carmine staining.

### 2.2. Methods

#### 2.2.1. Collection of General Data

**Figure 2:**
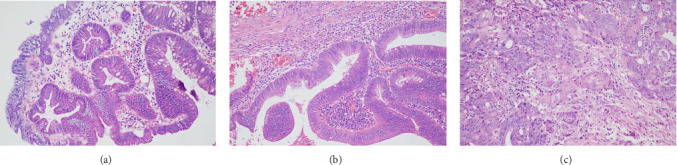
Pathological section of LSTs with a Skirt stained with HE. (a) Low-grade intraepithelial neoplasia. (b) High-grade intraepithelial neoplasia. (c) Cancer.

Gender, age, endoscopic features of LSTs (lesion diameter, location, morphology, presence of skirt), and histopathological classification were collected. Age was categorized into younger adults (< 60 years) and elderly patients (≥ 60 years). Lesion diameter refers to the longest axis of the lesion. Lesion location was classified as proximal colon (cecum, ascending colon, transverse colon), distal colon (descending colon, sigmoid colon), or rectum. Lesion morphology types included granular homogeneous, nodular mixed, flat elevated, and pseudodepressed. Histopathological types were categorized as hyperplastic polyps, adenomas with low-grade intraepithelial neoplasia (LGIN), adenomas with high-GIN (HGIN), and carcinoma (Figure 2).

### 2.3. Statistical Analysis

Statistical analysis was performed using SPSS 25.0 software. Normally distributed continuous data are expressed as mean ± standard deviation (*x* ± *s*). Independent sample *t*-tests were used for comparisons between two groups. Ranked data were analyzed using rank sum tests. Categorical data are presented as numbers and percentages, with comparisons between groups performed using Pearson's chi-squared test or Fisher's exact probability test. Logistic regression analysis was used to evaluate the impact of various factors on the presence of skirt lesions and the carcinogenesis of LSTs. *p* < 0.05 was considered statistically significant.

## 3. Results

### 3.1. Clinical and Pathological Characteristics of LSTs

A total of 390 LSTs cases were included in this study, with a mean age of 58.45 ± 12.00 years (221 males, 169 females). Lesions were located in the proximal colon (183 cases), distal colon (87 cases), and rectum (120 cases). The morphological distribution of LSTs was granular homogeneous (47 cases), nodular mixed (168 cases), flat elevated (139 cases), and pseudodepressed (36 cases). The diameter of the lesions was less than 20 mm in 208 cases and ≥ 20 mm in 182 cases. Histopathological analysis revealed 74 cases of hyperplastic polyps, 181 cases of adenomas with LGIN, 74 cases of adenomas with HGIN, and 64 cases of carcinoma. Details are shown in Table 1.

### 3.2. Comparison of Clinical and Pathological Features Between LSTs With and Without Skirt

Of the 390 LSTs cases, 30 were identified as LSTs with a skirt, with an incidence rate of 7.69%. Among the LSTs with a skirt, the mean age was 55.50 ± 10.85 years, with 19 females (63.33%), and 23 lesions (76.67%) located in the rectum. Twenty-six lesions (86.67%) had a diameter ≥ 20 mm. Histological types included no cases of hyperplastic polyps (0.00%), 10 cases of adenomas with LGIN (33.33%), 9 cases of adenomas with HGIN (30.00%), and 11 cases of carcinoma (36.67%). Among the 360 LSTs without a skirt, the mean age was 58.70 ± 12.07 years, with 150 female patients (41.67%) and 97 rectal lesions (26.94%). 156 lesions (43.33%) had a diameter ≥ 20 mm. Histopathological distribution included 74 hyperplastic polyps (20.56%), 172 adenomas with LGIN (47.5%), 62 adenomas with HGIN (17.22%), and 53 carcinomas (14.72%). The proportion of female patients, rectal lesions, lesions with a diameter ≥ 20 mm, nodular mixed morphology, and carcinoma in the LSTs with a skirt group was significantly higher than in the LSTs without a skirt group (*p* < 0.05). After excluding 8 patients who underwent additional surgical interventions, a total of 336 patients with LSTs completed postoperative follow-up, comprising 28 patients in the LSTs with a skirt group and 308 in the LSTs without a skirt group. The follow-up period ranged from 6 to 18 months, with a median duration of 11 months. The recurrence rate was significantly higher in the LSTs with a skirt group (10.71% vs. 1.62%; *p* < 0.05). Among the LSTs with a skirt group, 3 patients experienced local recurrence, two of whom underwent EMR, and one underwent ESD. Recurrence occurred in one case at 6 months, another at 8 months, and a third at 14 months postoperatively. All recurrent lesions were confirmed by biopsy as adenomas with low-grade dysplasia. Detailed results are shown in Table 2.

### 3.3. Factors Influencing the Presence of Skirt Lesions in LSTs

Logistic regression analysis showed that gender, lesion morphology, and histopathological classification were not independent factors for the occurrence of skirt lesions in colorectal LSTs. However, rectal lesions (*p*=0.001, OR = 8.588, 95% CI: 2.428–30.379) and lesion diameter ≥ 20 mm (*p*=0.008, OR = 4.538, 95% CI: 1.477–13.940) were independent predictive factors for the presence of skirt lesions in colorectal LSTs (Table 3).

### 3.4. Factors Influencing Carcinogenesis of LSTs With a Skirt

The cancer incidence rate of LSTs with a skirt in different groups is shown in Table 4. Logistic regression analysis revealed that older age (*p* = 0.002, OR = 22.667, 95% CI: 3.140–163.629) was an independent risk factor for the carcinogenesis of LSTs with a skirt (Table 5).

## 4. Discussion

Colorectal LSTs represent a distinct subtype of colorectal adenomas with a high potential for malignancy. Due to their unique growth pattern, LSTs are often missed during diagnosis, increasing the risk of submucosal invasive cancer [5]. Skirt lesions, which may serve as precursors to LSTs, contribute to their development. A study by Japanese researchers demonstrated that skirt lesions can undergo significant pathological changes within 3 months, including the expansion of round pits into III-L type pits and the appearance of mucosal capillaries, signaling a tendency for malignancy. Consequently, failure to detect or adequately remove skirt lesions during endoscopic procedures may elevate the risk of local recurrence of LSTs [6]. Therefore, investigating the occurrence and carcinogenic potential of LSTs with a skirt holds important clinical value.

In this study, 30 cases of skirt lesions were primarily identified using magnifying endoscopy or indigo carmine staining. Indigo carmine staining and magnifying endoscopy are effective in identifying skirt lesions. Under white light endoscopy, marginal lesions appear as slightly elevated, flat lesions with wide pits, occasionally observed at the margin of LSTs. In addition, magnifying endoscopy often reveals three types of pit patterns in the margin area: a coral reef-like appearance, a type II-like pit pattern, and a type III-L-like pit pattern. The surface microvasculature in this region typically displays a type I capillary pattern [7]. Indigo carmine staining enhances the contrast between the mucosal folds and surrounding tissue, facilitating clear visualization of flat, level lesions in the colon. This technique is more effective than routine nonstaining colonoscopy in detecting small, flat, elevated, and depressed lesions [8] and is particularly helpful for identifying skirt lesions.

Due to their subtle appearance and potential for malignancy, skirt lesions present a diagnostic challenge for endoscopists. Thus, understanding the unique clinical and pathological features that distinguish LSTs with a skirt from LSTs without a skirt is crucial for improving the detection rate of skirt lesions. In this study, potential factors influencing the presence of skirt lesions in LSTs included female gender, rectal location, nodular mixed-type lesions, lesion diameter ≥ 20 mm, and carcinoma. Age did not appear to affect the occurrence of skirt lesions. Previous studies have shown that LSTs may progress to HGIN, with an incidence ranging from 20.9% to 33.8% [9]. In patients with LSTs measuring ≥ 20 mm in diameter, the carcinoma transformation rate may reach up to 31% [10]. Therefore, early detection and resection of colorectal LSTs can significantly reduce both the incidence and mortality of colorectal cancer. Compared to LSTs without a skirt, LSTs with a skirt exhibited a lower incidence of LGIN but a higher incidence of HGIN and carcinoma, consistent with previous findings (20% adenomas, 80% carcinomas) [7]. This suggests that LSTs with a skirt may have a higher malignant potential. Among these factors, rectal location and lesion size ≥ 20 mm were identified as independent predictors of skirt lesions in LSTs, aligning with the results of Shozo Osera and colleagues [7]. Therefore, for LSTs with lesions ≥ 2 cm or located in the rectum, clinicians should be particularly attentive to the potential presence of skirt lesions. Therefore, when LSTs with skirt lesions are detected, we may consider lowering the intervention threshold for the skirt lesions. Even if the lesion is small, we recommend early and complete endoscopic resection, especially for lesions with unclear borders. In such cases, we suggest using indigo carmine staining or magnified endoscopy in advance to observe and accurately assess the nature and boundaries of the lesion, to avoid missing the skirted area during resection. Additionally, for lesions that have not yet been treated, a strict follow-up plan should be established (with endoscopic re-examination every 3–6 months).

Understanding the factors contributing to the carcinogenesis of LSTs is valuable for predicting the malignant potential of these lesions. Risk factors for carcinogenesis in colorectal LSTs include lesion size, location, morphology, and signs of elevation [11, 12]. However, findings from different studies are inconsistent. A multicenter study in Japan, which analyzed 1236 LSTs with diameters ≥ 20 mm, found a significant association between the risk of developing T1 colorectal cancer and pseudodepressed-type LSTs with larger diameters [13]. Lesion location and size varied across different LSTs morphologies. For instance, granular-type LSTs were most commonly found in the rectum and tended to have larger diameters. Li et al. [10] research indicated that larger lesion diameters, rectal location, and nodular mixed-type lesions are associated with an increased likelihood of malignancy in LSTs, especially the cancerization rate of LSTs was 31% in patients with tumor diameter ≥ 20 mm. Kyeong et al. [14] conducted a study involving 326 cases of LSTs and found that larger lesion diameters were associated with a higher incidence of malignant tumors. The incidence of malignancy was particularly elevated in the pseudodepressed type (24.3%) and the mixed nodular type (14.1%). Additionally, 16% of pseudodepressed lesions exhibited submucosal infiltration, a proportion significantly higher than that observed in other subtypes.

This study further revealed that the likelihood of malignancy in LSTs with a skirt is not associated with the lesion's location, size, or morphology, but is instead linked to the patient's age. Aging is a well-established risk factor for colorectal cancer. Miyamoto et al. [15] observed that LSTs with a skirt are more frequently found in the granular nodular mixed-type LSTs in the rectum, and that these cases are more prone to malignant transformation and submucosal infiltration. In a 2016 retrospective study by Osera Shozo et al. [7] 1023 patients with LSTs were included, of whom 35 were identified as having skirt-like structures. Among these 35 lesions, 80% contained intramucosal or submucosal adenocarcinoma components. In this study, pathological classifications in LSTs with a skirt patients aged over 60 were more likely to indicate malignancy. This suggests that clinicians should be particularly vigilant for malignancy in elderly patients who have LSTs with a skirt and conduct more comprehensive preoperative assessments.

In this study, the recurrence rate for LSTs without a skirt was 1.62%, consistent with previous reports [16] (the recurrence rate for ESD is 1%–2%). However, the postoperative recurrence rate for LSTs with a skirt was significantly higher than that for LSTs without a skirt (10.71% vs. 1.62%). Shozo Osera and colleagues have demonstrated that LSTs with a skirt have a significantly higher local recurrence rate after endoscopic treatment compared to LSTs without a skirt, particularly following EPMR. The risk of recurrence is closely associated with the presence of a skirt and piecemeal resection [17]. Therefore, preoperative identification of the lesion's skirt margin is crucial. It is recommended to perform indigo carmine and magnified endoscopy prior to resection to precisely delineate the lesion's extent. Furthermore, to achieve a complete resection, ESD should be the preferred technique, avoiding methods like EMR or EPMR, as ESD offers a higher overall resection rate, higher R0 resection rate, and lower risk of postoperative recurrence [18]. However, our study has limitations. It is a single-center, retrospective study with a relatively short follow-up period, which limits the long-term data. Future research should expand the sample size, extend the follow-up duration, and incorporate a multicenter prospective design to further confirm our findings.

When LSTs with skirt lesions are detected, we may lower the intervention threshold for the lesion. Even for small lesions, early complete endoscopic resection is recommended. For lesions with unclear boundaries, we suggest using indigo carmine staining or magnifying endoscopy to accurately assess the lesion's nature and borders. This approach helps avoid missing the skirted margin during resection, which could lead to positive resection margins. During postoperative follow-up, magnifying endoscopy with biopsy can be used to assess any residual lesions. Furthermore, patients with LSTs and a skirt who have not undergone endoscopic treatment, a rigorous follow-up plan should be implemented (colonoscopy every 3–6 months), and endoscopic treatment should be encouraged to improve the patient's prognosis.

This study is the first Chinese investigation to explore the clinical and pathological characteristics of LSTs with a skirt and their independent risk factors for malignant transformation, providing valuable insights for clinicians in recognizing and managing these lesions. In the conclusion, it is crucial to emphasize endoscopic management recommendations for elderly patients with large rectal LSTs (≥ 20 mm), such as the routine use of chromoendoscopy and magnifying techniques to improve lesion characterization and resection precision.

## Figures and Tables

**Table 1:** Clinical and pathological characteristics of overall LSTs.

Characteristics		
Sex, *n* (%)	Male	221 (56.67)
	Female	169 (43.33)
Age (year)		58.45 ± 12.00
Focal site, *n* (%)	The proximal colon	183 (46.92)
	The distal colon	87 (22.31)
	Rectum	120 (30.77)
LSTs type, *n* (%)	Granular homogeneous	47 (12.05)
	Nodular mixed	168 (43.08)
	Flat elevated	139 (35.64)
	Pseudodepressed	36 (9.23)
Diameter of the lesion, *n* (%)	< 20 mm	208 (53.33)
	≥ 20 mm	182 (46.67)
Histological classification, *n* (%)	Hyperplastic polyp	74 (18.97)
	LGIN	181 (46.41)
	HGIN	74 (18.97)
	Carcinoma	64 (16.41)

**Table 2:** Comparison of clinical and pathological characteristics between LSTs with a skirt and LSTs without a skirt.

	LSTs with a skirt (*n* = 30)	LSTs without a skirt (*n* = 360)	*t*/*χ*^2^	*p*
Sex, *n* (%)			5.294	0.021
Male	11 (36.67)	210 (58.33)		
Female	19 (63.33)	150 (41.67)		
Age (year)	55.50 ± 10.85	58.70 ± 12.07	1.405	0.161
Focal site, *n* (%)			32.867	< 0.001
The proximal colon	3 (10.00)	180 (50.00)		
The distal colon	4 (13.33)	83 (23.06)		
Rectum	23 (76.67)	97 (26.94)		
LSTs type, *n* (%)			23.109	< 0.001
Granular homogeneous	4 (13.33)	43 (11.94)		
Nodular mixed	24 (80.00)	144 (40.00)		
Flat elevated	1 (3.33)	138 (38.33)		
Pseudodepressed	1 (3.33)	35 (9.72)		
Diameter of the lesion, *n* (%)			20.893	< 0.001
< 20 mm	4 (13.33)	204 (56.67)		
≥ 20 mm	26 (86.67)	156 (43.33)		
Histological classification, *n* (%)			18.835	< 0.001
Hyperplastic polyp	0 (0.00)	74 (20.56)		
LGIN	10 (33.33)	171 (47.50)		
HGIN	9 (30.00)	62 (17.22)		
Carcinoma	11 (36.67)	53 (14.72)		
Recurrence, *n* (%)	3/28 (10.71)	5/308 (1.62)	5.634	0.018

**Table 3:** Multivariate analysis results of colorectal LSTs with a skirt.

	*β*	Wals	*p*	OR	95% CI
Female			0.087		
Rectum	2.150	11.129	0.001	8.588	2.428–30.379
Diameter of the lesion ≥ 20 mm	1.512	6.977	0.008	4.538	1.477–13.940
Nodular mixed			0.065		
Carcinoma			0.731		

**Table 4:** The incidence rate of cancer in different groups of LSTs with a skirt based on various factors.

	Noncancerous	Cancerous
Sex, *n* (%)		
Male	6 (31.58)	5 (45.45)
Female	13 (68.42)	6 (54.55)
Age, *n* (%)		
Middle-aged and young	17 (89.47)	3 (27.27)
Elderly	2 (10.53)	8 (72.73)
Focal site, *n* (%)		
Nonrectum	6 (31.58)	1 (9.09)
Rectum	13 (68.42)	10 (90.91)
LSTs type, *n* (%)		
Granular homogeneous	3 (15.79)	1 (9.09)
Nodular mixed	15 (78.95)	9 (81.82)
Flat elevated	1 (5.26)	0 (0.00)
Pseudodepressed	0 (0.00)	1 (9.09)
Diameter of the lesion, *n* (%)		
< 20 mm	3 (15.79)	1 (9.09)
≥ 20 mm	16 (84.21)	10 (90.91)

**Table 5:** Logistic regression analysis results of colorectal LSTs with a skirt for carcinogenesis.

	*β*	Wals	*p*	OR	95% CI
Female			0.918		
Elderly	3.121	9.576	0.002	22.667	3.140–163.629
Rectum			0.115		
Nodular mixed			0.806		
Diameter of the lesion ≥ 20 mm			0.531		

## Data Availability

The data that support the findings of this study are available on request from the corresponding author. The data are not publicly available due to privacy or ethical restrictions.

## References

[1] Danpanichkul P., Suparan K., Tothanarungroj P. (2024). Epidemiology of Gastrointestinal Cancers: a Systematic Analysis from the Global Burden of Disease Study 2021. *Gut*.

[2] Lu S., Jia C. Y., Yang J. S. (2023). Future Therapeutic Implications of New Molecular Mechanism of Colorectal Cancer. *World Journal of Gastroenterology*.

[3] Lin Y., Zhang X., Li F. (2025). A Deep Neural Network Improves Endoscopic Detection of Laterally Spreading Tumors. *Surgical Endoscopy*.

[4] Miyamoto H., Oono Y., Fu K. L. (2013). Morphological Change of a Laterally Spreading Rectal Tumor over a Short Period. *BMC Gastroenterology*.

[5] Castillo-Regalado E., Uchima H. (2022). Endoscopic Management of Difficult Laterally Spreading Tumors in Colorectum. *World Journal of Gastrointestinal Endoscopy*.

[6] Okamoto T., Tanaka S., Haruma K. (1996). [Clinicopathologic Evaluation on Colorectal Laterally Spreading Tumor (LST)]. *Nihon Shokakibyo Gakkai Zasshi*.

[7] Osera S., Fujii S., Ikematsu H. (2016). Clinicopathological, Endoscopic, and Molecular Characteristics of the “skirt”: A New Entity of Lesions at the Margin of Laterally Spreading Tumors. *Endoscopy (Stuttgart)*.

[8] Saito Y., Ono A., García V. A. J. (2021). Diagnosis and Treatment of Colorectal Tumors: Differences between Japan and the West and Future Prospects. *DEN Open*.

[9] Kim B. C., Chang H. J., Su Han K. (2011). Clinicopathological Differences of Laterally Spreading Tumors of the Colorectum According to Gross Appearance. *Endoscopy (Stuttgart)*.

[10] Li D. H., Liu X. Y., Huang C. (2021). Pathological Analysis and Endoscopic Characteristics of Colorectal Laterally Spreading Tumors. *Cancer Management and Research*.

[11] Hao X. W., Li P., Wang Y. J., Ji M., Zhang S. T., Shi H. Y. (2022). Predictors for Malignant Potential and Deep Submucosal Invasion in Colorectal Laterally Spreading Tumors. *World Journal of Gastrointestinal Oncology*.

[12] Tanaka S, Kashida H, Saito Y (2020). Japan Gastroenterological Endoscopy Society Guidelines for Colorectal Endoscopic Submucosal Dissection/Endoscopic Mucosal Resection. *Digestive Endoscopy*.

[13] Tanaka S., Kashida H., Saito Y. (2020). Japan Gastroenterological Endoscopy Society Guidelines for Colorectal Endoscopic Submucosal Dissection/endoscopic Mucosal Resection. *Digestive Endoscopy*.

[14] Kim K. O., Jang B. I., Jang W. J., Lee S. H. (2013). Laterally Spreading Tumors of the Colorectum: Clinicopathologic Features and Malignant Potential by Macroscopic Morphology. *International Journal of Colorectal Disease*.

[15] Miyamoto H., Ikematsu H., Fujii S. (2014). Clinicopathological Differences of Laterally Spreading Tumors Arising in the Colon and Rectum. *International Journal of Colorectal Disease*.

[16] Belderbos T. D., Leenders M., Moons L. M., Siersema P. D. (2014). Local Recurrence after Endoscopic Mucosal Resection of Nonpedunculated Colorectal Lesions: Systematic Review and Meta-Analysis. *Endoscopy (Stuttgart)*.

[17] Osera S., Ikematsu H., Fujii S. (2017). Endoscopic Treatment Outcomes of Laterally Spreading Tumors with a Skirt (With Video). *Gastrointestinal Endoscopy*.

[18] Alric H., Barret M., Becar A. (2024). Endoscopic Submucosal Dissection versus Endoscopic Mucosal Resection for Laterally Spreading Rectal Tumours. *Colorectal Disease: The Official Journal of the Association of Coloproctology of Great Britain and Ireland*.

